# Acute hemiplegia: aetiology and outcome in Nigerian children

**DOI:** 10.11604/pamj.2020.36.155.20594

**Published:** 2020-07-06

**Authors:** Komomo Eyong, Chimaeze Torty, Asindi Asindi, Emmanuel Ekanem

**Affiliations:** 1 Department of Paediatrics, University of Calabar Teaching Hospital, Calabar, Nigeria

**Keywords:** Acute hemiplegia, aetiology, outcome

## Abstract

**Introduction:**

acute hemiplegia of childhood is a postnatally acquired nonspecific clinical response of the brain to various aetiological insults in a child who was neurologically normal at birth. This study aims at evaluating the aetiology and outcome of acute hemiplegia in children admitted into the University of Calabar Teaching Hospital (UCTH), Nigeria.

**Methods:**

a 5-year retrospective review of all children admitted to the Neurology Unit of the Department of Paediatrics of UCTH with a diagnosis of acute hemiplegia. The demographic characteristics of the children and the clinical features were noted. Investigations including neuroimaging of the brain and haemoglobin genotype were documented. The outcomes of the patients were recorded as either dead, recovered with deficit or loss to follow up. Data obtained was analysed using the SPSS version 24. Simple tables were used to display the results in number and percentages.

**Results:**

twenty-five children with diagnosis of hemiplegia were admitted. Associated clinical features were prolonged seizures (68%), speech defect (32%), cranial nerve deficit (36%) and loss of consciousness (12%). Viral encephalitis was the common aetiology in 11(44%) of the patients, followed by meningitis and sickle cell anaemia in 6(24%) patients each. Four(16%) of the patients recovered completely within the follow up period of three month, 19(76%) had varying degrees of weakness; 2(8%) died. Twelve (48%) were lost to follow-up.

**Conclusion:**

central nervous system infections and sickle cell disease as dominant aetiological factors of acute hemiplegia in Nigerian children. This calls for effective infection control and genetic counselling.

## Introduction

Acute hemiplegia of childhood describes a postnatally acquired nonspecific clinical response of the brain to various aetiological insults in a child who was neurologically intact at birth. This excludes children with hemiplegia related to perinatal insult such as prematurity, birth asphyxia, birth trauma and perinatal infection. Hemiplegia is less common in children compared to adults and the pathological condition and underlying aetiologies thereof are much more varied [1,2]. Cerebrovascular event or stroke is the most common cause of acute hemiplegia in adult whereas in children a number of other conditions such as CNS infection (e.g. encephalitis, meningitis and abscess), neoplastic intracranial space-occupying lesions (ICSOL), trauma and developmental anomalies of the brain are major contributing factors [1-4]. During the latter part of 19^th^century, Freud described acute hemiplegia of childhood as a condition in which a previously healthy child (aged few months to 3 years) without hereditary predisposition suddenly becomes ill; the aetiology of the illness was unknown or is sought in a simultaneously occurring infectious disease [5,6]. With the advent of more sophisticated investigation modalities such as cerebral angiography only a few are of unknown cause [7]. This study is aimed at determining the aetiology and outcome of children admitted with acute hemiplegia in a tertiary hospital in a developing country.

## Methods

The study is a 5-year retrospective review on the records of all children admitted to the Children Emergency and the Neurology Unit of the Department of Paediatrics of the University of Calabar Teaching Hospital UCTH, Calabar, Nigeria, with a diagnosis of acute hemiplegia. These includes all children with hemiplegia of acute onset (weakness on one side of the body) that occurred postnatally and excludes hemiplegia arising from perinatal events such as prematurity, severe birth asphyxia, birth trauma and CNS infections. The demographic characteristics of the children abstracted from the record were age, gender, ethnic group, the social class of the children. The premorbid conditions of the children, particularly those with sickle cell anaemia (HbSS), were noted. Children with cerebral palsy and chronic hemiplegia were excluded from the study. As a hospital policy, all children presenting with neurological symptoms including hemiplegia are admitted into the neurology wing of the paediatric ward. The unit is manned by three consultant neurologists and residents. There are no facilities for viral studies/serology. Patients were presumed to have viral encephalitis if they were admitted with high fever, convulsions, altered sensorium plus other features of acute cerebral symptomatology but their spinal fluid was normal and malaria smear was negative. The clinical features at presentation were noted; and laboratory investigations undertaken during the acute events including blood chemistry, full blood count, haemoglobin genotype, spinal fluid analysis, malaria smear and brain neuroimaging were recorded. The outcomes of the patients were recorded as either dead, recovered with deficit or lost to follow up. All clinical information obtained was entered into a Microsoft Excel spread sheet and same analysed using the SPSS version 24. Simple tables were used to display the results in number and percentages.

## Results

Within the 5-year period of study, 25 children with a diagnosis of acute hemiplegia were admitted into the neurology unit. These were made of 13 males and 12 females. The age/gender distributions of the children are shown in Table 1. In 15(60%) of the children the hemiplegia left-sided while 10(40%) were right-sided. Associated clinical features included prolonged seizures in 17 (68%) of patients, speech defect in 8(32%), cranial nerve deficit in 9(36%) and loss of consciousness in 3(12%) of the cases. Thirteen (52%) of the patients presented with level III of gross motor function classification system (GMFCS) while 8(32%) and 4(16%) of the patients belong to level II and IV respectively. Viral encephalitis is the commonest identified aetiology for acute hemiplegia in 11(44%) of the patients, this is followed by meningitis and haemoglobinopathy (HbSS) and meningitis in 6(24%) each (Table 2). Of the 25 patients recruited in the study, 4(16%) recovered completely without any gross motor deficit within three months, 19(76%) recovered with varying degree of weakness and 2(8%) died. Twelve (48%) patients were lost to follow up within three months. Children with GMFCS levels of I and II had improved survival compared to those in levels III-V. Computerized tomographic scan on some of the patients shows hemispheric cerebral atrophy ex-vacou dilatation of the ipsilateral ventricle.

**Table 1:** age and sex distribution of 25 children with acute hemiplegia

	Sex		
Age	Males	Females	Total
<5	4	6	10
6-10	4	3	7
>10	5	3	8
Total	13	12	25

**Table 2:** aetiology of hemiplegia in 25 children

Aetiology	Frequency	Percentages
Viral encephalitis^**^	11	44
Meningitis	6	24
Sickle cell anaemia	6	24
CNS tumours	2	8
Total	25	100

** Three of these were related to HIV encephalopathy

## Discussion

From this study, CNS infections comprising of viral encephalitis and meningitis appear to be the dominant underlying causes of acute hemiplegia in Nigerian children. Other similar surveys [4-6,8-10] have also shown that infections accounted for between 15 to 56% of the aetiology of acute hemiplegia in children. Central nervous system infections are associated with local vasculitis and thrombosis leading to hemiplegia [8]. In this study this related vascular phenomenon could have accounted for the over 50% of the cases of acute hemiplegia. Hemiplegia occurs during the course of bacterial meningitis as a consequence of vasculitis or venous thrombosis. During the course of viral encephalitis, especially that involving herpes simplex, there is parenchymal necrosis. In both bacterial and viral infections, prolonged or repetitive focal seizures tend to precede the hemiplegia. A multicenter study of Nigerian sickle cell patients with stroke documented a prevalence of 74 per cent [11] which is higher than the 24% prevalence seen in our study. This variation in the prevalence may be as a result of the small number of patients in our study. The pathophysiology of hemiplegia in sickle cell disease remains poorly understood and probably varies with the site of vascular injury.

Increased red cell adhesion, oxidative injury of the vessel wall, inflammation, abnormal vasomotor tone regulation and increased activity of the coagulation system may all contribute to cerebral vascular obstruction in sicklers [12]. Three (12%) of the children who developed hemiplegia had HIV infection. Two main pathologies are thought to be responsible for focal neurologic deficits in any patient with HIV infection; these are cerebral toxoplasmosis (typically presenting with headache, confusion and fever in addition to focal neurological deficit in varying degrees) and CNS lymphomas which occur with increased lifespan of HIV-infected patients [13,14]. The outcome of acute hemiplegia is highly variable and depends on the underlying cause [15]. Four of the children in this study with acute hemiplegia recovered without any neurological deficit. Possibly due to our small sample size, there was no statistically significant relationship between the aetiology and the outcome, however, children with GMFCS levels of I and II had improved survival compared to those in levels III-V. Data available from other studies on children with arterial ischemic stroke during follow up indicate that 30% were neurologically normal, about two third had motor or cognitive deficit and mortality was 9% [15] which is comparable to the 8% documented in our study.

## Conclusion

Infections of the CNS and sickle cell anaemia appear to be the predominant underlying causes of acute hemiplegia of childhood in Nigeria. It is therefore necessary that every child with acute hemiplegia should be evaluated for CNS infections and sickle cell disease. Infectious disease control and genetic counselling regarding sickle cell hemoglobinopathy, can be useful regarding the prevention of this handicapping condition in children.

### What is known about this topic

- Acute hemiplegia occur in children;
- The aetiology of acute hemiplegia varies from that of adults;
- Heamoglobinopathies are common causes of acute hemiplegia in children.

### What this study adds

- Central nervous system infections should be considered as an important differential in all cases acute hemiplegia;
- HIV infection should considered as an important cause of acute hemiplegia in children and routine screening should be carried out in this condition;
- Sickle cell heamoglobinothies still remains an impotant cause in the tropics.
